# CircRNA expression profiles and functional analysis in a mouse model of chronic intermittent hypoxia-induced renal injury: new insight into pathogenesis

**DOI:** 10.7717/peerj.14957

**Published:** 2023-02-28

**Authors:** Qingshi Chen, Zhenzhen Hong, Zhiyu Chen, Yanfeng Chen, Dexin Liu

**Affiliations:** 1 Department of Endocrinology and Metabolism, The Second Affiliated Hospital of Fujian Medical University, Quanzhou, China; 2 Department of Radiology, The Second Affiliated Hospital of Fujian Medical University, Quanzhou, China

**Keywords:** Obstructive sleep apnea, circRNAs, Chronic intermittent hypoxia, Renal injury, Microarray

## Abstract

Increasing evidence has demonstrated that circular RNAs (circRNAs) play crucial roles in the pathogenesis of multiple diseases. However, the functions of circRNAs in renal injury induced by obstructive sleep apnea (OSA) are poorly understood. The aim of this current study is to identify the global changes of circRNAs expression in OSA-induced renal damage. The mouse model of OSA treated by chronic intermittent hypoxia (CIH) was established. We assessed the expression profiles of circRNAs in CIH caused renal injury by microarray analysis. Bioinformatic analyses were further performed by us to assess those differentially expressed circRNAs. Quantitative realtime PCR (qRT-PCR) were then conducted to assure the data of microarray. Finally, a circRNA-miRNA -mRNA competing endogenous RNA (ceRNA) regulatory network was constructed. We found 11 upregulated and 13 downregulated circRNAs in CIH-induced renal injury. The qRT-PCR validated that the six selected circRNAs were identical to the results of microarray. Both Gene ontology (GO) and Kyoto Encyclopedia of Genes and Genomes (KEGG) analysis were further employed to annotate the potential functions of dysregulated circRNAs. Finally, we established a ceRNA network to predict the target genes of circRNAs. In general, our results first illustrate that circRNAs are aberrantly expressed in OSA-induced renal injury, which might aid in offering novel genetic insights into this disease and potential therapeutic targets for OSA-associated chronic kidney disease.

## Introduction

Obstructive sleep apnea (OSA) is well-known as one of the most common sleep‑disordered breathing, which affects millions of people globally (Xia & Sawan, 2021). Now, OSA has been recognized as one of the significant risk factor for type 2 diabetes (Gabryelska et al., 2020), asthma (Oka et al., 2020), stroke (Ponsaing et al., 2018), preeclampsia–eclampsia (Jaimchariyatam et al., 2019), and cardiovascular diseases (Gottlieb & Punjabi, 2020). Meanwhile, OSA has been regarded as the important causes of various renal injury (Loffler et al., 2017). Increasing evidence indicates that severe OSA patients have a high prevalence of chronic kidney disease (CKD). OSA has further been shown to be responsible for accelerated loss of kidney function (Rimke et al., 2021). It is reported that OSA-associated CKD is mostly caused by chronic intermittent hypoxia (CIH) triggered tissue damage. Abuyassin et al. (2018) found histopathological alterations of kidney in response to OSA induced by intermittent hypoxia in mouse models. However, the underlying mechanism of OSA-induced renal injury remains unclear.

Circular RNAs (circRNAs) are a novel type of endogenous non-coding RNAs, which are identified with covalently closed loop structures without 5′ to 3′ polarity and a poly A tail (Zhang, Liu & Liao, 2020; Yang et al., 2021). CircRNAs are widely expressed in different species, and exhibit tissue/developmental stage-specificity. For instance, hsa_circ_0000517 was found to harbor HCC-stage-specific expression features in hepatocellular carcinoma (Wang et al., 2019). Thus, it suggests that circRNAs could serve as a novel diagnostic biomarker for various diseases. Recent studies have showed that circRNAs are aberrantly expressed in human diseases and exert their functions *via* regulation of target genes at the post-transcriptional level (Zeng et al., 2021). As reported, hsa_circ_001680 influences the proliferation and migration of colorectal carcinoma by targeting miR-340 to regulate the expression of BMI1 (Jian et al., 2020). Presently, accumulating evidence demonstrates that circRNAs are closely associated with certain human diseases, such as diabetic cardiomyopathy (Wan et al., 2021), acute myeloid leukemia (Lux et al., 2021), osteoarthritis (Wu et al., 2021b), and several types of cancers (Rong et al., 2021; Fan et al., 2021; Liu et al., 2021). Nevertheless, little is known about the roles of circRNAs in renal damage induced by OSA at present.

In the present study, we assessed the circRNA expression profiles to gain an overview of the expression patterns of circRNAs in mouse models of OSA-induced renal injury. Subsequently, we further used qRT-PCR to validate six candidate circRNAs. Additionally, we performed a bioinformatics analysis to predict the biological functions of the abnormally expressed circRNAs. Finally, we further constructed a circRNA-miRNA-mRNA (ceRNA) interaction network to reveal the potential mechanisms of five selected dysregulated circRNAs. Thus, through these explorations, we aimed to provide a novel insight into the progress of OSA-related CKD and assist management of this disease in clinical practice.

## Materials and Methods

### Mouse model of CIH

We bought three pairs of male BABL/c mice from Beijing Weitong Lihua Experimental Animal Technology co., ltd. We randomly divided the mice into two groups: the control group and the CIH group. We provided all mice with tap water and standard mouse diet. The standard CIH protocol was modeled as described in our previous study (Chen et al., 2019). We placed mice assigned to receive CIH in a specially designed chamber, which contained oxygen sensors for measuring the concentration of O_2_ in the chamber. Cages were connected to a gas regulator that controls the infusion of sufficient nitrogen to reduce O_2_ to 6% for 60 s, after which the gas control system allows for a rapid replacement of oxygen leading to a quick restoration of O_2_ to 21% for another 60 s. This 2-min CIH cycle was repeated 30 times per h, 8 h/day, for a total duration of 8 weeks. Their kidney tissues were acquired after euthanized by cervical dislocation at the end of CIH exposure. Animal procedures were complied with the ARRIVE 2.0 guidelines. The animal protocol was approved by the Animal Care and Use Committee of the Second Affiliated Hospital of Fujian Medical University (2022-FMU-15).

### Microarray analysis

Following the Arraystar standard protocols, we first carried out sample preparation and microarray hybridization. In brief, we digested the total RNA of renal tissues with RNase R (Epicentre, Inc., Madison, WI, USA) aiming to remove linear RNAs and enrich circRNAs. After that, we amplified and transcribed the enriched circRNAs into fluorescent circRNAs with the use of a random priming method (Arraystar Super RNA Labeling kit; Arraystar, Rockville, MD, USA). Subsequently, we hybridized the labeled circRNAs to the Arraystar Mouse circRNA Array V2 (8x15K; Arraystar, Rockville, MD, USA). After washing the slides, an Agilent Scanner G2505C was further used to scan the arrays. Then, we utilized Agilent Feature Extraction software (version 11.0.1.1) to analyze the acquired array images. We also conducted quantile normalization and subsequent data processing by the use of R software package. Finally, we identified the differentially expressed circRNAs between the CIH group and the control group through Volcano Plot filtering. At the same time, a scatter plot and heat map were used by us to evaluate the variation of circRNAs expression between the two groups.

### Bioinformatic analyses

To functionally annotate parental genes of the dysregulated circRNAs, Gene Ontology (GO) analyses were further carried out in our study, which included cellular component (CC), molecular function (MF) and biological process (BP). Further, KEGG pathway analyses were used to reveal the signaling networks of circRNAs associated with OSA-induced renal injury.

### qRT-PCR analysis

We performed qRT-PCR analysis of six circRNAs to validate the results of the microarray data. Briefly, the total RNA derived from the renal tissue samples was reverse-transcribed into complementary DNA (cDNA) with the help of SuperScript™ III Reverse Transcriptase (18080-044; Invitrogen, Waltham, MA, USA). According to the manufacturer’s instructions, 2X PCR Master Mix (AS-MR-006-5; Arraystar, Rockville, MD, USA) and ViiA 7 Real-Time PCR system (Applied Biosystems, Waltham, MA, USA) were used for qRT-PCR. The PCR primer sequences were outlined in Table 1. GAPDH was employed to serve as the internal control. Target gene expression was analyzed by the 2^−ΔΔCt^ equation.

**Table 1:** Primers used for qRT-PCR.

circRNA ID	Primers	Tm (° C)	Product size (bp)
GAPDH	F: 5′ CACTGAGCAAGAGAGGCCCTAT3′ R: 5′ GCAGCGAACTTTATTGATGGTATT3′	60	144
mmu_circRNA_35869	F: 5′ ACACTGACATCGAGGGCATAGA3′ R: 5′GAGCAGGAGGGATTGTAGAAAAC3′	60	122
mmu_circRNA_27795	F: 5′ GCCGGGAAACAATTAGAGG 3′ R: 5′ CAGAAGGCTCGTTCAGGATG3′	60	214
mmu_circRNA_35632	F: 5′ GGAAGTTTGGCAGCAGAATC3′ R: 5′ CAATTCCGTAAGTCTGTGCATC3′	60	203
mmu_circRNA_009555	F: 5′ AGTTCCACGTACTGTTCCTTT 3′ R: 5′ CTTGCTGTCTTTCTTGGCTGA3′	60	172
mmu_circRNA_29626	F: 5′ GGACATCGTGGAGTGGTTGAA 3′ R: 5′ AGAGACCTTTGTGGAGGACAGC 3′	60	63
mmu_circRNA_37351	F: 5′ TTGAGCCGAAGCCAGAGGA 3′ R: 5′ CGAGGTTGGTGGTCAGGAAA 3′	60	153

### Construction of a ceRNA regulatory network

Finally, the interaction between the five candidate circRNAs and their potential target microRNAs was mainly predicted by miRanda and TargetScan software. Then, a ceRNA interaction network was constructed, which was based on the prediction of miRNA binding sites. The established ceRNA network was further visualized by use of Cytoscape (Version 3.7.2).

### Statistical methods

All data in the present study were obtained from at least three independent experiments. Data were presented as mean ± SD and compared by using Student’s t-test between the CIH and control groups. Two-tailed *P* < 0.05 was considered significant.

## Results

### Changes in the expression profiles of circRNAs

**Figure 1:**
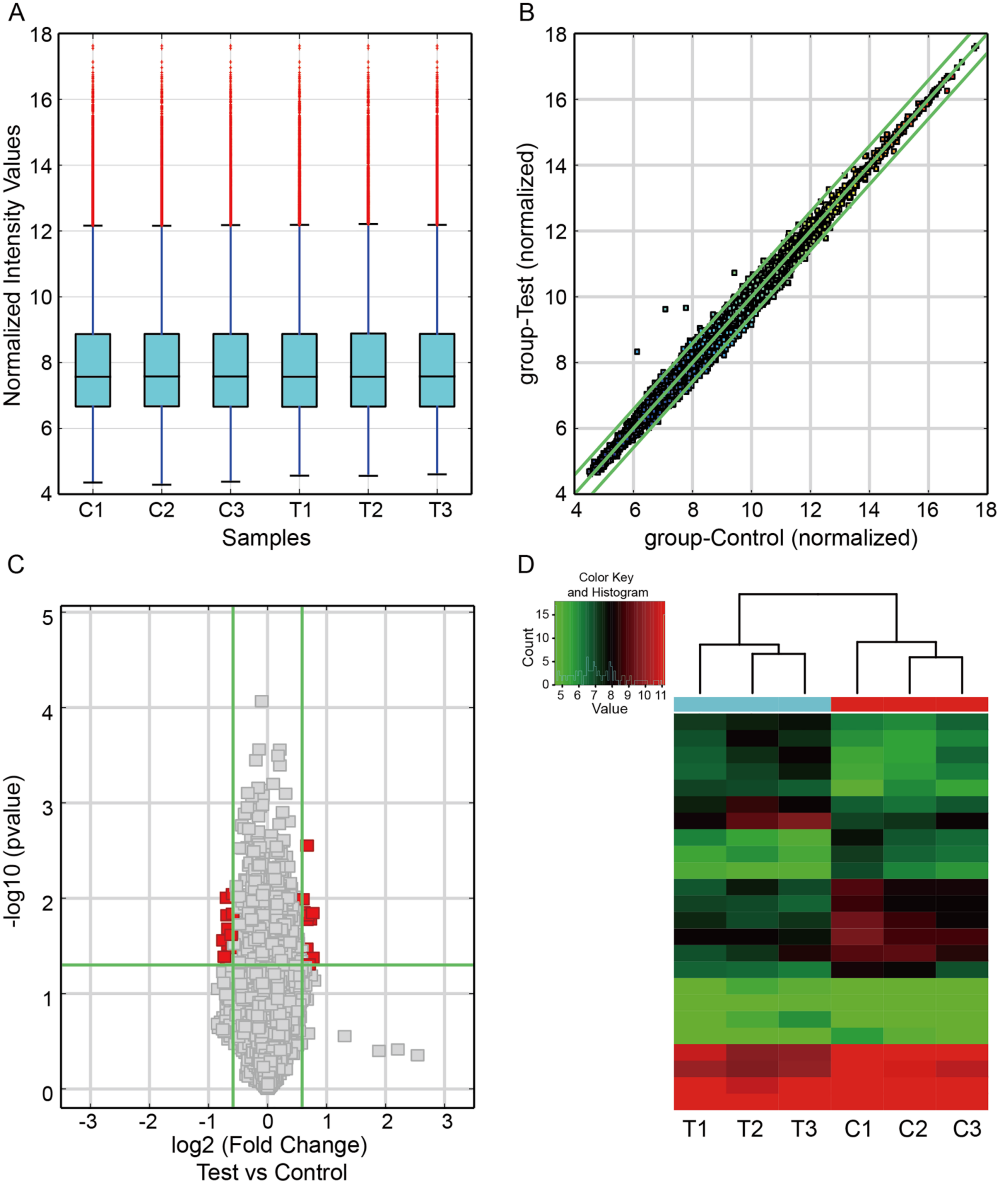
Differentially expressed circRNAs in renal from CIH mice. (A) The box plot demonstrated the nearly identical distributions of circRNAs in the renal samples of the CIH and Control groups. The red data points are outliers. (B) The scatter plot was used to identify the variation of circRNAs expression between the two groups. The green lines represented 1.5-fold changes. circRNAs beyond the range of green lines indicated >1.5-fold difference between the two compared groups. (C) The volcano plot exhibited significantly aberrant circRNAs in CIH-induced renal injury. The red squares represented the dysregulated circRNAs with significant difference. (D) Hierarchical clustering analysis revealed a distinct circRNA expression pattern between the two groups.

To investigate the circRNA expression profiles in CIH-induced renal injury, we carried out the circRNA microarray to identify the dysregulated circRNAs. The box plot showed the distributions of circRNAs expression profiles from three paired renal samples was not different (Fig. 1A). A scatter plot was utilized to visualize circRNA expression variations between the two groups (Fig. 1B). The volcano plot filter indicated differentially altered circRNAs with log2FC ≥1.5 and *P* value < 0.05 (Fig. 1C). Hierarchical clustering showed a distinguishable expression profile of circRNAs among the samples (Fig. 1D). Finally, we observed that 24 circRNAs were differentially expressed in this study, including 13 downregulated and 11 upregulated circRNAs in renal damage induced by CIH.

**Figure 2:**
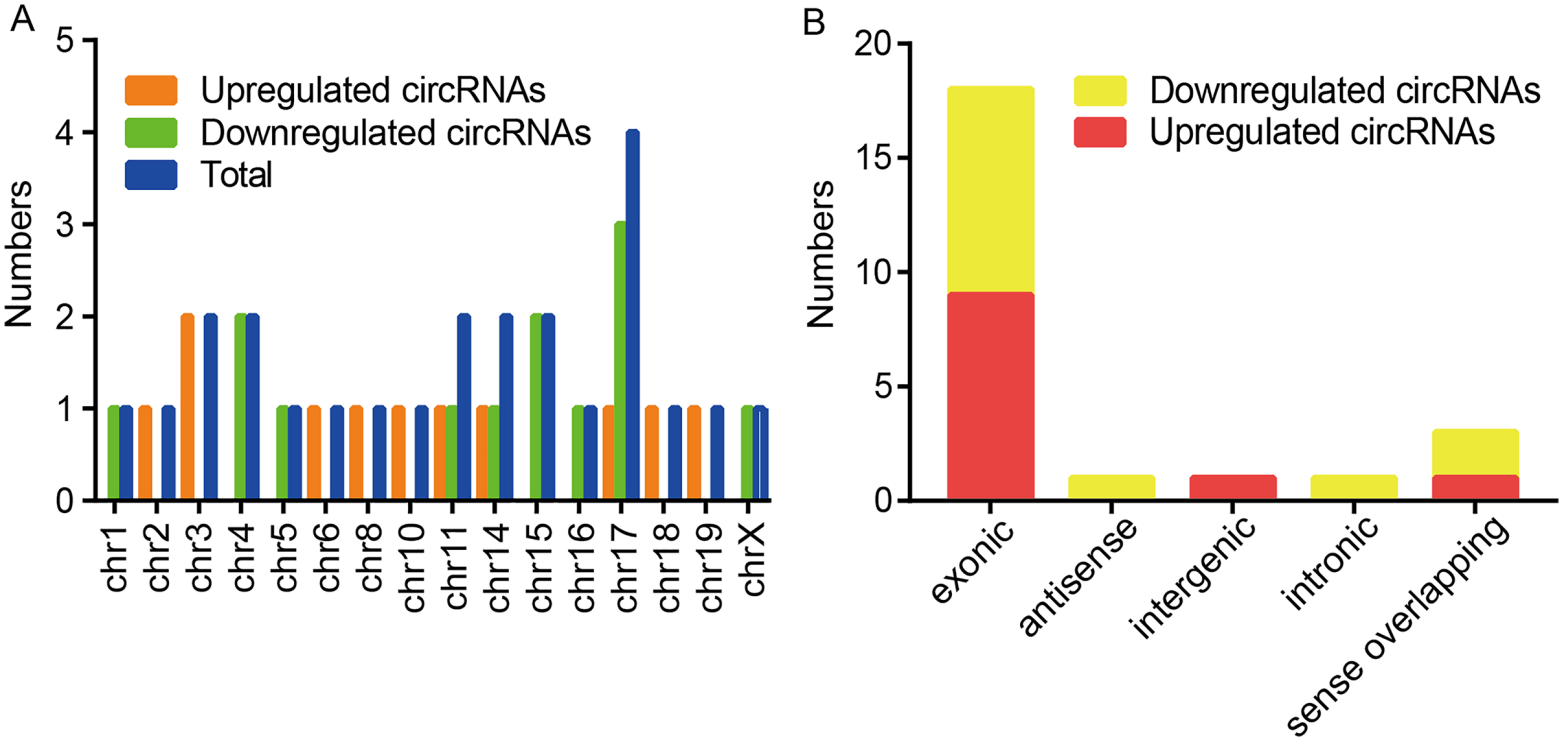
Characteristics of altered circRNAs. (A) Chromosomal distribution of the dysregulated circRNAs between the CIH and Control groups. (B) Classification of the significantly altered circRNAs between the two groups.

Among these dysregulated circRNAs, they were widely distributed on almost all mouse chromosomes, and chromosome 17 was the most abundant (Fig. 2A). The properties of circRNAs contain exonic, antisense, sense overlapping, intronic, and intergenic. The compositional type of each sample is shown in Fig. 2B. For the upregulated circRNAs, they contained nine exonic circRNAs, one intergenic circRNA, and one sense overlapping circRNA. For the downregulated circRNAs, they included nine exonic circRNAs, one antisense circRNA, one intronic circRNA and two sense overlapping circRNAs.

### Validation of six selected dysregulated circRNAs

**Figure 3:**
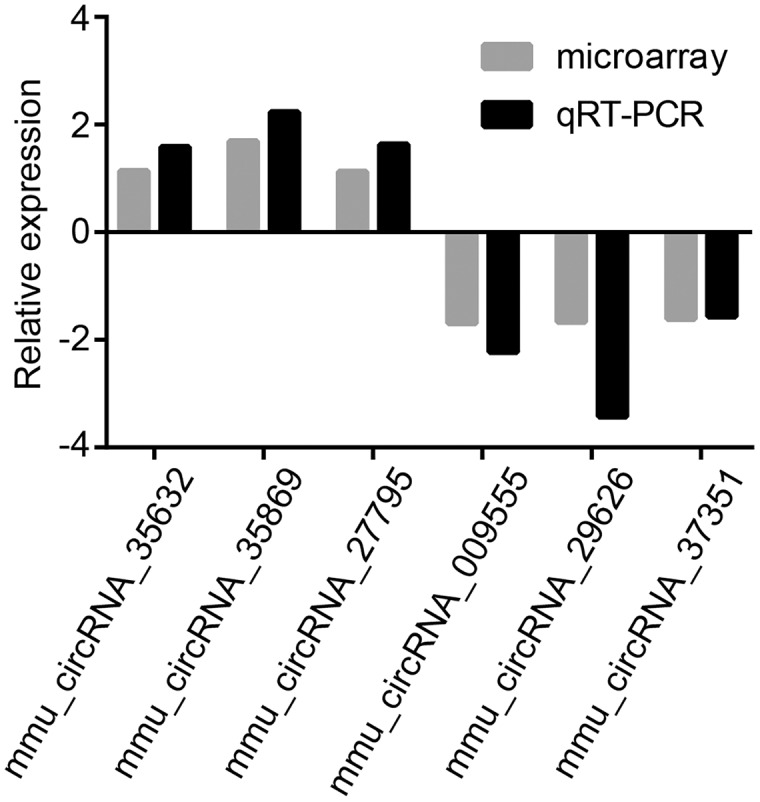
Validation of dysregulated circRNAs. The relative expression levels of six selected circRNAs were determined by qRT-PCR. The upward direction represents gene upregulation. On the contrary, downward direction represents gene downregulation.

To validate microarray results, six circRNAs (mmu_circRNA_35869, mmu_circRNA_27795, mmu_circRNA_35632, mmu_circRNA_009555, mmu_circRNA_29626 and mmu_circRNA_37351) were randomly selected for further evaluation by qRT-PCR analysis. The expression levels of them were in accordance with that of circRNA microarray data (Fig. 3). GAPDH served as the internal control.

### GO and KEGG analyses

**Figure 4:**
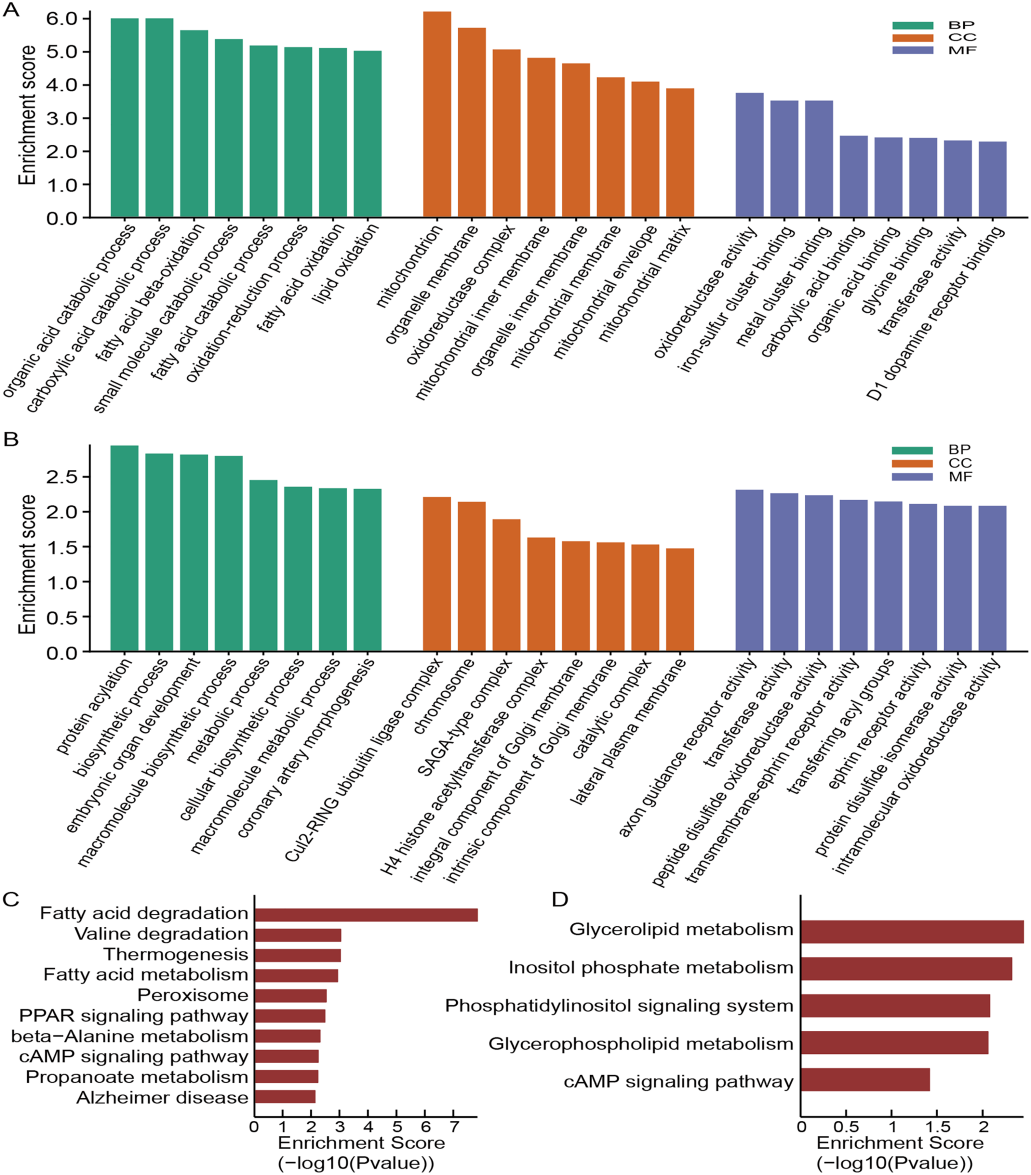
Functional analysis of altered circRNAs. GO enrichment analysis for upregulated (A) and downregulated (B) circRNAs. KEGG analysis of upregulated (C) and downregulated (D) circRNAs.

To annotate the function of these dysregulated circRNAs, we then conducted the GO and KEGG analysis. The top eight enriched GO categories were listed as a bar plot. GO enrichment analysis of the upregulated circRNAs indicated the most enriched GO terms to be organic acid catabolic process (BP), oxidoreductase activity (MF) and mitochondrion (CC) (Fig. 4A), while the most enriched GO entries for downregulated circRNAs were protein acylation (BP), axon guidance receptor activity (MF) and Cul2-RING ubiquitin ligase complex (CC) (Fig. 4B). Intriguingly, our findings showed that the upregulated circRNAs involved in CC category were connected with the mitochondrion, mitochondrial inner membrane, organelle membrane, mitochondrial membrane, oxidoreductase complex, mitochondrial matrix, organelle inner membrane, and mitochondrial envelope, which demonstrated that mitochondrial impairment might play a key role in the process of OSA-induced renal injury.

According to the KEGG pathway analysis, the most relevant and enriched pathway for the upregulated circRNAs was the fatty acid degradation (Fig. 4C), while the downregulated circRNAs were significantly enriched in the Glycerolipid metabolism (Fig. 4D). In addition, we also found that PPAR signaling pathway and cAMP signaling pathway may also participate in the pathophysiological process of OSA-induced renal damage.

### Establishment of ceRNA network

**Figure 5:**
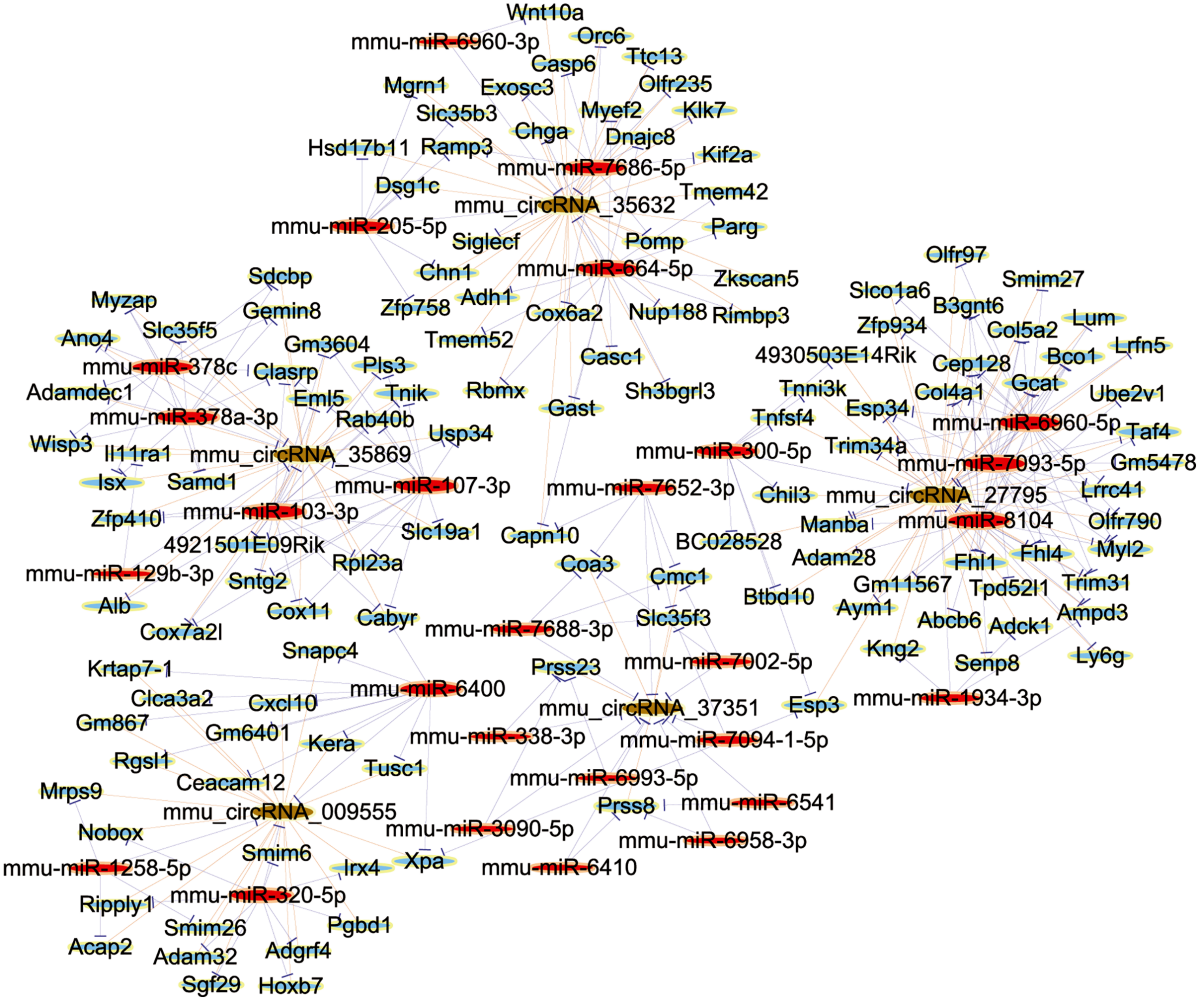
The ceRNA Network Construction. Light-blue nodes represent protein_coding RNAs, red nodes represent miRNAs, light-green nodes are noncoding RNAs, and brown nodes represent circRNAs.

In order to elucidate the ceRNA network, we predicted the targets of differentially expressed circRNAs and their downstream-regulated genes by the use of TargetScan and miRanda. Further, to explore the bio-function of circRNAs involved in renal damage induced by OSA, we made use of Cytoscape software (Version 3.7.2; https://cytoscape.org/) to build up a ceRNA network, which was based on the combinatorial effect of five selected circRNAs (mmu_circRNA_35869, mmu_circRNA_27795, mmu_circRNA_35632, mmu_circRNA_009555 and mmu_circRNA_37351), and their potential miRNA targets and downstream-regulated mRNAs (Fig. 5). This information provided a significant clue for us to reveal the molecular mechanisms of circRNA in the renal injury triggered by OSA. In summary, circRNAs may act as a competitive endogenous RNA in the OSA-induced kidney injury by sponging to multiple miRNAs. As a marked putative ceRNA, mmu_circRNA_35869 will be investigated in our further studies.

## Discussion

In the study, we first performed a comprehensive analysis of circRNAs by using microarray assay in renal injury induced by OSA. The results showed that 24 circRNAs were differentially expressed in the mouse model. Among these dysregulated circRNAs, 11 of them were upregulated and 13 were down-regulated. Moreover, the expression levels of the six selected circRNAs were verified by qRT-PCR analyses, which were consistent with the microarray. Finally, we conducted GO and KEGG analyses and constructed a ceRNA regulatory network to predict the potential role of circRNAs in the process of OSA-induced renal damage. These findings could provide novel clues to reveal the role of circRNAs in the pathologic mechanisms of OSA related CKD.

Several reports showed that CIH was associated with cardiovascular diseases (Yan et al., 2021a), hepatic injuries (Lin et al., 2020), and pulmonary injury (Ding et al., 2021). It was also reported that CIH could lead to chronic kidney disease (Zhao et al., 2021). Accumulating evidences suggest that OSA might contribute to various renal diseases *via* its association with increased sympathetic nervous system activity, endothelial dysfunction, inflammation, renin-angiotensin-aldosterone system overactivation, and increased oxidative stress (Abuyassin et al., 2015; Rimke et al., 2019). The prevalence of OSA in patients of CKD is several folds higher than that in general population. Meanwhile, the frequency of CKD also increases with the severity of OSA. The treatment of OSA improves the prognosis of patients with CKD. For example, Nicholl et al. (2021) reported high rate of kidney dysfunction occurred in patients who have OSA. Treatment of OSA patients with the CPAP therapy causes a decrease in glomerular hyperfiltration (Nicholl et al., 2021). In our study, we established a CIH mouse model and found that CIH could induce renal structure impairment. Therefore, we confirmed that CIH participated in the pathological changes of renal damage caused by OSA. However, the underlying mechanism responsible for the initiation and progression of OSA-induced renal injury remains unclear.

Recently, given the crucial roles of circRNAs in various biological and disease processes, these molecules have gained a tremendous amount of attention among researchers. As reported, circRNAs have been found implicated with various diseases, including autoimmune diseases (Xia, Tang & Wang, 2019), lung cancer (Wang et al., 2020), and renal disease (Jin et al., 2020). To explore the potential functions of circRNAs in OSA-induced injury in kidney, we conducted a microarray analysis of circRNAs in a mouse model of CIH. In this study, we identified a total of 24 differentially expressed circRNAs. Among them, six randomly selected circRNAs, including mmu_circRNA_35869, mmu_circRNA_27795, mmu_circRNA_35632, mmu_circRNA_009555, mmu_circRNA_29626 and mmu_circRNA_37351, were testified by qRT-PCR. The expression of the six selected circRNAs were confirmed in accordance with the microarray analysis. Above all, our results revealed that that a large number of circRNAs show significant differential expression in the pathogenesis of CIH triggered renal damage. However, the function and mechanism of these circRNAs are largely unknown.

To further explore the regulative roles of circRNAs in OSA-induced renal injury, both GO and KEGG analysis were carried out for the functional annotation of parental genes. GO analysis of the up-regulated circRNAs illuminated that the significant enrichment of cellular components was tightly related to mitochondrial impairment, such as mitochondrion, organelle membrane, mitochondrial inner membrane, mitochondrial membrane, mitochondrial envelope, and mitochondrial matrix. Previous studies showed that mitochondrial impairment mainly results in cell injuries in kidney diseases (Schijvens et al., 2020; Shamekhi Amiri, 2019). Moreover, recent studies suggested that CIH could cause the damage of target organs through its mitochondrial regulation (Wu et al., 2021a; Yan et al., 2021b). Thus, we make a conclusion that the dysfunction of mitochondrial may play an important role in the transcriptional regulation of OSA-induced renal injury. Further experimental researches are highly needed to explore the potential function of mitochondrial impairment in renal injury trigged by OSA.

Although the function of circRNAs remains largely unknown, their function as a miRNA sponge has been well documented. Here, circRNAs could act as sponges, bind to their target miRNAs, inhibit the transcription of mRNA, and then further influence the pathophysiological processes of various diseases (Panda, 2018; Liang et al., 2020). The construction of ceRNA networks is an effective method to explore the roles of circRNAs. To investigate the downstream regulatory genes, we selected five circRNAs to perform ceRNA analyses after qRT-PCR verification. Our results indicated that the five selected circRNAs were found to have extensive interactions with multiple miRNAs. These findings suggested that circRNAs could sponge to miRNAs to regulate the expression of targe genes, which could add a new dimension of our current knowledge on OSA-induced renal injury pathogenesis. Among the ceRNA interaction network, mmu_circRNA_35869 and mmu_circRNA_35632 harbored a putative binding site with miR-107-3p and miR-205-5p, respectively. To our interest, it was reported that miR-107 could target to DUSP7 inducing the secretion of TNF-α in endothelial cells, which directly contributed to tubular cell injury in septic acute kidney injury (Wang et al., 2017). In addition, miR-205 improved renal cell apoptosis *via* the HMGB1-PTEN pathway, which might provide a new target for the therapy of sepsis-induced renal injury (Zhang et al., 2019). These data demonstrated that the mmu_circRNA_35869/miR-107-3p and mmu_circRNA_35632/miR-205-5p axis may play critical roles in the progression of renal injury induced by OSA. Thus, further studies are in urgent need to confirm the functions of the circRNA-related ceRNA network in OSA-induced renal damage.

Several limitations in our study should not be neglected. First, since it was a relatively small sample size study, meaning that we should take cautions to interpret the results. Second, we predicted all the functional annotation of the differentially expressed circRNAs based only on bioinformatics analysis. Further experimental investigations are needed for clarifying the underlying mechanisms and function of the present circRNAs, such as *in vivo* and *vitro* experiments. Third, there were some important differences between our animal model and OSA model in humans. The animal model of OSA eliminates many potential confounders, which were commonly found in human studies. Herein, further clinical researches are still needed in future.

## Conclusions

In conclusion, this work first reveals the comprehensive expression profiles of circRNAs in a mouse model of OSA-induced renal injury, which expands the current understanding in the pathogenesis mechanism of renal damage induced by OSA. Therefore, these findings suggest a potential treatment of OSA-related chronic kidney disease through the modulation of circRNAs. Deciphering the molecular mechanism about the detailed role the circRNAs identified in this study requires further investigation.

## Supplemental Information

10.7717/peerj.14957/supp-1 Supplemental Information 1 ARRIVE 2.0 Checklist.

10.7717/peerj.14957/supp-2 Supplemental Information 2 Raw data for qRT-PCR (Figure 3).

10.7717/peerj.14957/supp-3 Supplemental Information 3 MIAME checklist.

## Additional Information and Declarations

### Data Availability

The following information was supplied regarding data availability: The data is available at NCBI GEO: GSE192688.

## References

[ref-1] Abuyassin B, Badran M, Ayas NT, Laher I, Joles JA (2018). Intermittent hypoxia causes histological kidney damage and increases growth factor expression in a mouse model of obstructive sleep apnea. PLOS ONE.

[ref-2] Abuyassin B, Sharma K, Ayas NT, Laher I (2015). Obstructive sleep apnea and kidney disease: a potential bidirectional relationship?. Journal of Clinical Sleep Medicine.

[ref-3] Chen Q, Lin G, Huang J, Chen G, Huang X, Lin Q (2019). Expression profile of long non-coding RNAs in rat models of OSA-induced cardiovascular disease: new insight into pathogenesis. Sleep and Breathing.

[ref-4] Ding W, Zhang X, Zhang Q, Dong Y, Wang W, Ding N (2021). Adiponectin ameliorates lung injury induced by intermittent hypoxia through inhibition of ROS-associated pulmonary cell apoptosis. Sleep and Breathing.

[ref-5] Fan C, Qu H, Xiong F, Tang Y, Tang T, Zhang L, Mo Y, Li X, Guo C, Zhang S, Gong Z, Li Z, Xiang B, Deng H, Zhou M, Liao Q, Zhou Y, Li X, Li Y, Li G, Wang F, Zeng Z (2021). CircARHGAP12 promotes nasopharyngeal carcinoma migration and invasion via ezrin-mediated cytoskeletal remodeling. Cancer Letters.

[ref-6] Gabryelska A, Karuga FF, Szmyd B, Białasiewicz P (2020). HIF-1α as a mediator of insulin resistance, T2DM, and its complications: potential links with obstructive sleep apnea. Frontiers in Physiology.

[ref-7] Gottlieb DJ, Punjabi NM (2020). Diagnosis and management of obstructive sleep apnea. JAMA.

[ref-8] Jaimchariyatam N, Na-rungsri K, Tungsanga S, Lertmaharit S, Lohsoonthorn V, Totienchai S (2019). Obstructive sleep apnea as a risk factor for preeclampsia–eclampsia. Sleep and Breathing.

[ref-9] Jian X, He H, Zhu J, Zhang Q, Zheng Z, Liang X, Chen L, Yang M, Peng K, Zhang Z, Liu T, Ye Y, Jiao H, Wang S, Zhou W, Ding Y, Li T (2020). Hsa_circ_001680 affects the proliferation and migration of CRC and mediates its chemoresistance by regulating BMI1 through miR-340. Molecular Cancer.

[ref-10] Jin J, Sun H, Shi C, Yang H, Wu Y, Li W, Dong YH, Cai L, Meng XM (2020). Circular RNA in renal diseases. Journal of Cellular and Molecular Medicine.

[ref-11] Liang ZZ, Guo C, Zou MM, Meng P, Zhang TT (2020). circRNA-miRNA-mRNA regulatory network in human lung cancer: an update. Cancer Cell International.

[ref-12] Lin ZP, Lin HL, Yu XP, Zheng YJ, Cheng SY (2020). TLR4 mediates inflammation and hepatic fibrosis induced by chronic intermittent hypoxia in rats. Molecular Medicine Reports.

[ref-13] Liu J, Jiang X, Zou A, Mai Z, Huang Z, Sun L, Zhao J (2021). circIGHG-induced epithelial-to-mesenchymal transition promotes oral squamous cell carcinoma progression via miR-142-5p/IGF2BP3 signaling. Cancer Research.

[ref-14] Loffler KA, Heeley E, Freed R, Anderson CS, Brockway B, Corbett A, Chang CL, Douglas JA, Ferrier K, Graham N, Hamilton GS, Hlavac M, McArdle N, McLachlan J, Mukherjee S, Naughton MT, Thien F, Young A, Grunstein RR, Palmer LJ, Woodman RJ, Hanly PJ, McEvoy RD (2017). Effect of obstructive sleep apnea treatment on renal function in patients with cardiovascular disease. American Journal of Respiratory and Critical Care Medicine.

[ref-15] Lux S, Blätte TJ, Gillissen B, Richter A, Cocciardi S, Skambraks S, Schwarz K, Schrezenmeier H, Döhner H, Döhner K, Dolnik A, Bullinger L (2021). Deregulated expression of circular RNAs in acute myeloid leukemia. Blood Advances.

[ref-16] Nicholl DDM, Hanly PJ, Zalucky AA, Handley GB, Sola DY, Ahmed SB (2021). Nocturnal hypoxemia severity influences the effect of CPAP therapy on renal renin-angiotensin–aldosterone system activity in humans with obstructive sleep apnea. Sleep.

[ref-17] Oka S, Goto T, Hirayama A, Faridi MK, Camargo CA, Hasegawa K (2020). Association of obstructive sleep apnea with severity of patients hospitalized for acute asthma. Annals of Allergy, Asthma & Immunology.

[ref-18] Panda AC (2018). Circular RNAs act as miRNA sponges. Advances in Experimental Medicine and Biology.

[ref-19] Ponsaing LB, Lindberg U, Rostrup E, Iversen HK, Larsson HBW, Jennum P (2018). Impaired cerebrovascular reactivity in obstructive sleep apnea: a case-control study. Sleep Medicine.

[ref-20] Rimke AN, Ahmed SB, Turin TC, Pendharkar SR, Raneri JK, Lynch EJ, Hanly PJ (2019). Effect of CPAP therapy on kidney function in patients with obstructive sleep apnoea and chronic kidney disease: a protocol for a randomised controlled clinical trial. BMJ Open.

[ref-21] Rimke AN, Ahmed SB, Turin TC, Pendharkar SR, Raneri JK, Lynch EJ, Hanly PJ (2021). Effect of CPAP therapy on kidney function in patients with chronic kidney disease. Chest.

[ref-22] Rong Z, Xu J, Shi S, Tan Z, Meng Q, Hua J, Liu J, Zhang B, Wang W, Yu X, Liang C (2021). Circular RNA in pancreatic cancer: a novel avenue for the roles of diagnosis and treatment. Theranostics.

[ref-23] Schijvens AM, van de Kar NC, Bootsma-Robroeks CM, Cornelissen EA, van den Heuvel LP, Schreuder MF (2020). Mitochondrial disease and the kidney with a special focus on CoQ10 deficiency. Kidney International Reports.

[ref-24] Shamekhi Amiri F (2019). Intracellular organelles in health and kidney disease. Néphrologie & Thérapeutique.

[ref-25] Wan H, Zhao S, Zeng Q, Tan Y, Zhang C, Liu L, Qu S (2021). CircRNAs in diabetic cardiomyopathy. Clinica Chimica Acta.

[ref-26] Wang C, Tan S, Li J, Liu W, Peng Y, Li W (2020). CircRNAs in lung cancer—biogenesis, function and clinical implication. Cancer Letters.

[ref-27] Wang X, Wang X, Li W, Zhang Q, Chen J, Chen T (2019). Up-regulation of hsa_circ_0000517 predicts adverse prognosis of hepatocellular carcinoma. Frontiers in Oncology.

[ref-28] Wang S, Zhang Z, Wang J, Miao H (2017). MiR-107 induces TNF-α secretion in endothelial cells causing tubular cell injury in patients with septic acute kidney injury. Biochemical and Biophysical Research Communications.

[ref-29] Wu X, Gong L, Xie L, Gu W, Wang X, Liu Z, Li S (2021a). NLRP3 deficiency protects against intermittent hypoxia-induced neuroinflammation and mitochondrial ROS by promoting the PINK1-parkin pathway of mitophagy in a murine model of sleep apnea. Frontiers in Immunology.

[ref-30] Wu Y, Hong Z, Xu W, Chen J, Wang Q, Chen J, Ni W, Mei Z, Xie Z, Ma Y, Wang J, Lu J, Chen C, Fan S, Shen S (2021b). Circular RNA circPDE4D protects against osteoarthritis by binding to miR-103a-3p and regulating FGF18. Molecular Therapy.

[ref-31] Xia F, Sawan M (2021). Clinical and research solutions to manage obstructive sleep apnea: a review. Sensors.

[ref-32] Xia X, Tang X, Wang S (2019). Roles of CircRNAs in autoimmune diseases. Frontiers in Immunology.

[ref-33] Yan YR, Zhang L, Lin YN, Sun XW, Ding YJ, Li N, Li HP, Li SQ, Zhou JP, Li QY (2021a). Chronic intermittent hypoxia-induced mitochondrial dysfunction mediates endothelial injury via the TXNIP/NLRP3/IL-1β signaling pathway. Free Radical Biology and Medicine.

[ref-34] Yan YR, Zhang L, Lin YN, Sun XW, Ding YJ, Li N, Li HP, Li SQ, Zhou JP, Li QY (2021b). Chronic intermittent hypoxia-induced mitochondrial dysfunction mediates endothelial injury via the TXNIP/NLRP3/IL-1β signaling pathway. Free Radical Biology and Medicine.

[ref-35] Yang X, Ye T, Liu H, Lv P, Duan C, Wu X, Jiang K, Lu H, Xia D, Peng E, Chen Z, Tang K, Ye Z (2021). Expression profiles, biological functions and clinical significance of circRNAs in bladder cancer. Molecular Cancer.

[ref-36] Zeng X, Yuan X, Cai Q, Tang C, Gao J (2021). Circular RNA as an epigenetic regulator in chronic liver diseases. Cells.

[ref-37] Zhang Y, Liu Q, Liao Q (2020). CircHIPK3: a promising cancer-related circular RNA. American Journal of Translational Research.

[ref-38] Zhang Y, Xia F, Wu J, Yang AX, Zhang YY, Zhao H, Tao WY (2019). MiR-205 influences renal injury in sepsis rats through HMGB1-PTEN signaling pathway. European Review for Medical and Pharmacological Sciences.

[ref-39] Zhao L, Liu T, Dou Z, Wang M, Hu Z, Wang B (2021). CB1 receptor antagonist rimonabant protects against chronic intermittent hypoxia-induced renal injury in rats. BMC Nephrology.

